# Crosstalk between Signaling Pathways in Pemphigus: A Role for Endoplasmic Reticulum Stress in p38 Mitogen-Activated Protein Kinase Activation?

**DOI:** 10.3389/fimmu.2017.01022

**Published:** 2017-09-05

**Authors:** Gabriel A. Cipolla, Jong Kook Park, Robert M. Lavker, Maria Luiza Petzl-Erler

**Affiliations:** ^1^Department of Genetics, Federal University of Paraná, Curitiba, Brazil; ^2^CAPES Foundation, Ministry of Education of Brazil, Brasília, Brazil; ^3^Department of Dermatology, Feinberg School of Medicine, Northwestern University, Chicago, IL, United States; ^4^Department of Biomedical Science and Research, Institute for Bioscience and Biotechnology, Hallym University, Chuncheon, South Korea

**Keywords:** pemphigus, autoimmunity, p38 mitogen-activated protein kinase, endoplasmic reticulum stress, apoptolysis

## Abstract

Pemphigus consists of a group of chronic blistering skin diseases mediated by autoantibodies (autoAbs). The dogma that pemphigus is caused by keratinocyte dissociation (acantholysis) as a distinctive and direct consequence of the presence of autoAb targeting two main proteins of the desmosome—desmoglein (DSG) 1 and/or DSG3—has been put to the test. Several outside-in signaling events elicited by pemphigus autoAb in keratinocytes have been described, among which stands out p38 mitogen-activated protein kinase (p38 MAPK) engagement and its apoptotic effect on keratinocytes. The role of apoptosis in the disease is, however, debatable, to an extent that it may not be a determinant event for the occurrence of acantholysis. Also, it has been verified that compromised DSG trans-interaction does not lead to keratinocyte dissociation when p38 MAPK is inhibited. These examples of conflicting results have been followed by recent work revealing an important role for endoplasmic reticulum (ER) stress in pemphigus’ pathogenesis. ER stress is known to activate the p38 MAPK pathway, and *vice versa*. However, this relationship has not yet been studied in the context of activated signaling pathways in pemphigus. Therefore, by reviewing and hypothetically connecting the role(s) of ER stress and p38 MAPK pathway in pemphigus, we highlight the importance of elucidating the crosstalk between all activated signaling pathways, which may in turn contribute for a better understanding of the role of apoptosis in the disease and a better management of this life-threatening condition.

## Introduction

Pemphigus encompasses various chronic autoimmune blistering skin diseases with acute stages often controlled by the administration of glucocorticosteroid drugs, especially when co-administrated with an adjuvant (1). However, the lack of specificity and the broad effect of steroid treatment may impact patient’s homeostasis. The side effects of short- and long-term treatments with glucocorticosteroid drugs are well documented and may even include death [reviewed in Ref. (2–6)]. Successful clinical trials with more specific drugs have been reported, such as rituximab (anti-CD20) and protein A immunoadsorption (7, 8). However, our limited knowledge of the disease has hampered the development of highly specific therapeutic agents that would ultimately minimize the use of glucocorticosteroid drugs as therapeutic agents.

Historically, pemphigus is identified by pathogenic IgG autoantibodies (autoAbs) targeting adhesion molecules of keratinocytes. These autoantigens are mainly two desmosomal cadherins: desmoglein (DSG) 1 and/or DSG3. Two main forms of pemphigus usually associate to autoAb profiles (9–12). In pemphigus foliaceus (PF), typically only anti-DSG1 autoAb, superficial blistering and erosions are observed. In contrast, pemphigus vulgaris (PV) patients may exhibit anti-DSG3 or anti-DSG3 and anti-DSG1 autoAb, known to specify the suprabasal blistering of mucous membranes or a mucocutaneous form, respectively [reviewed in Ref. (13, 14)]. While the anti-DSG profiles are highly indicative of the clinical form, the observation that reduction of disease activity toward a remitting stage may be followed by the maintenance of high anti-DSG titers remains a conundrum (15). However, the molecular mechanisms that comprise anti-DSG autoAb and lead to the loss of adhesion between keratinocytes in pemphigus are a major puzzle. Here, we review what has been generally proposed for pemphigus’ pathogenesis, while suggesting a potentially p38 mitogen-activated protein kinase (p38 MAPK)-co-shared central role for endoplasmic reticulum (ER) stress in the disease.

## Conceivable Molecular Mechanisms for Loss of Cell Adhesion in Pemphigus

Two main explanations, the first one simpler and more intuitive and the latter more recently proposed, have been put together as our knowledge of the disease has evolved. The simpler model proposes that pemphigus pathogenic autoAb inhibit, either sterically or allosterically, the interaction of DSG1 and DSG3 from desmosomes of adjacent keratinocytes (trans-interaction), inducing loss of cell adhesion. Such hindrance would take place at the extracellular (EC) domains 1 and 2 located in the NH_2_-terminal region of DSGs, where pathogenic autoAb have been shown to bind specifically (16–20). Importantly, at least one of these EC domains is believed to allow for cis- and trans-interactions necessary for robust binding between adjacent keratinocytes (21).

In clinical terms, this model has been corroborated by the main findings that, in a Brazilian endemic form of PF known as *fogo selvagem*, patients in the preclinical stage exhibit IgG1 autoAb against the EC5 domain of DSG1, and that the onset of disease is accompanied by the emergence of IgG4 autoAb recognizing the EC1 and EC2 domains of the molecule (22). Similarly, mucosal PV is suggested to evolve to mucocutaneous PV upon intramolecular epitope spreading of autoAb against EC domains of the COOH-terminal region to autoAb against EC domains of the N-terminus portion of DSG3, as the former autoAb fail to recognize human skin and the latter autoAb have affinity for this tissue (23). This intramolecular epitope spreading in DSG3 is believed to precede an intermolecular epitope spreading from DSG3 to DSG1, an autoAb profile that correlates with the mucocutaneous form of PV (9). However, that autoAb against the EC C-terminus portion of DSG3 can be pathogenic themselves and that PV patients may have anti-DSG3-N-terminus portion autoAb without showing skin lesions suggest another layer of complexity to this model of pemphigus’ pathogenesis (23). The fact that some PF and PV patients also exhibit IgG or other isotypes of autoAb with specificity to different keratinocyte adhesion and/or non-adhesion molecules also argues in favor of a more complex pathogenesis [reviewed in Ref. (24, 25)]. Moreover, DSG1 and DSG3 have been shown to be targeted also by other immunoglobulin isotypes, specifically by IgE and IgM autoAb, which may as well play a role in disease development [reviewed in Ref. (26, 27)].

The rather simple explanation of pemphigus’ pathogenesis through steric hindrance relies on the DSG compensation hypothesis, which states that the distribution of DSG1 and DSG3 in the epidermis determines the site of blistering in pemphigus skin. Based on what has been discussed so far, this suggests that either DSG1 or DSG3 could solely account for epidermal cohesion. However, a new concept for the pathogenesis of pemphigus derives from a series of observations of signaling pathways activated by PF and PV autoAb [reviewed in Ref. (24)]. Among these activated pathways, researchers have described involvement of cyclic adenosine monophosphate (cAMP), epidermal growth factor receptor kinase (EGFRK), heat shock protein 27 (HSP27), c-Jun N-terminal kinases (JNK), mechanistic target of rapamycin, phospholipase C, protein kinases A and C, p38 MAPK, tyrosine-protein kinase SRC, and other tyrosine kinases (28–36). This new model has been termed apoptolysis, referring to the loss of epidermal cell adhesion (acantholysis) as a main outcome of the activation of keratinocyte intracellular apoptotic enzymes following the binding of distinctive autoAb profiles in pemphigus (36, 37).

It is not clear, however, whether apoptosis is a necessary preceding event for the pathognomonic acantholysis in pemphigus patients. In fact, in a large-scale electron microscopy study of pemphigus skin and mucosa, no cellular changes compatible with apoptosis were observed in lesional or non-lesional tissue (38). Moreover, molecular evidences have also argued against the role of apoptosis in blistering given the lack of consistent TUNEL positivity and detection of apoptotic markers, such as cleaved caspase 3, in pemphigus biopsies (39, 40). In a review on the topic, Schmidt and Waschke reported that most of the experiments suggesting a role for apoptosis in pemphigus were based on keratinocyte culture assays and their incubation with PV-derived IgG (41). This has been interpreted as a consequence of the high levels of Fas ligand present in pemphigus sera, which would be a trigger for the extrinsic apoptotic pathway (42). Independently of apoptosis being a primary or secondary, or even an irrelevant event in pemphigus’ pathogenesis, the signal transducing component of the apoptolytic theory is a well-supported and expected sequence of events, as illustrated below by the role of p38 MAPK pathway engagement in pemphigus. In summary, the concept of altered signaling involves the following events: (i) phosphorylation of adhesion or non-adhesion molecules with desmosome disassembly, (ii) derangement and collapse of the cytoskeleton, and (iii) impaired formation of new intercellular desmosomes and/or keratinocyte apoptosis [reviewed in Ref. (24, 41)].

**Figure 1:**
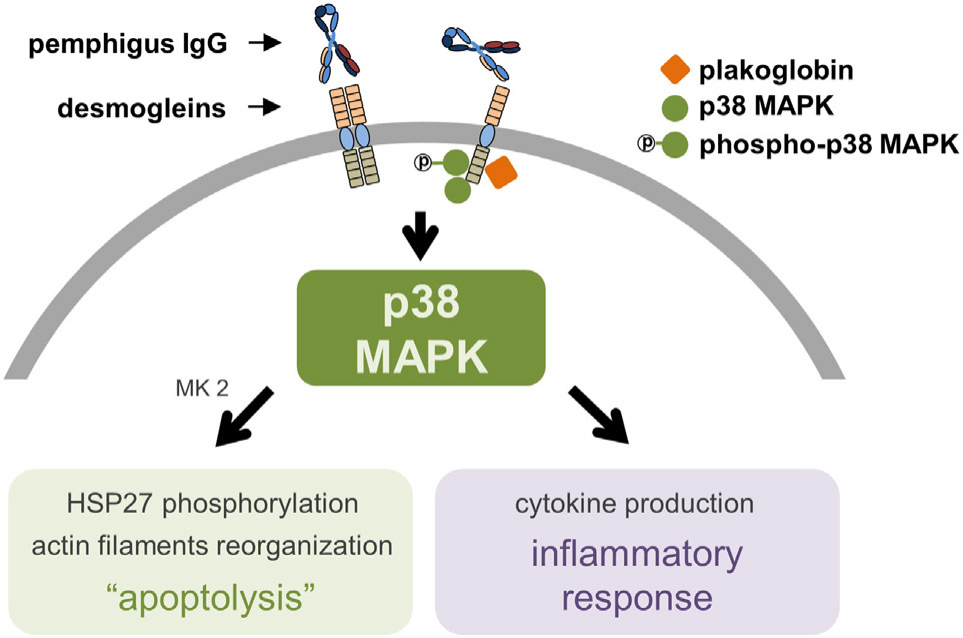
The proposed central role of p38 mitogen-activated protein kinase (p38 MAPK) in pemphigus’ pathogenesis. The molecular complex supposed to be formed by desmoglein (DSG, specifically DSG3, as of the gap for data on DSG1), plakoglobin, and p38 MAPK induces keratinocyte apoptolysis and a local inflammatory response after binding of pemphigus IgG to extracellular domains of DSG. A phosphorylation cascade involving p38 MAPK, MAPK activated protein kinase 2 (MK2), and heat shock protein 27 (HSP27) leads to the collapse of the cytoskeleton and ultimately to keratinocyte apoptosis. The central role of p38 MAPK includes the production of pro-inflammatory cytokines.

Although the apoptolysis hypothesis was initially proposed based on direct observations, as the DSG compensation hypothesis fails to explain the mismatch between the autoAb pattern/DSG1 and DSG3 distribution and the morphological blistering phenotype observed in PV (24), the apoptolytic mechanism considers several other events also observed in PF. Initially, cell culture and cell-free assays showed that anti-DSG1 autoAb derived from PF patients induce keratinocyte dissociation without direct inhibition of DSG1 trans-interaction, possibly requiring cell-dependent signaling mechanisms (43). Furthermore, PF IgG also activated the p38 MAPK pathway and induced blister formation in a murine model of PF (44). More recently, a careful inspection of the ultrastructure of PF lesional skin revealed: almost detached keratinocytes with severe stretching of plasma membranes in pre-acantholytic areas; desmosomes still attached but beginning to tear off from cell membranes; and full desmosomes torn off from keratinocytes (45). This would agree with the third and fourth steps of the apoptolytic mechanism, after transduction of apoptolytic signals from plasma membranes (step 1), and elevation of intracellular calcium and launching of cell death cascades (step 2): “collapse and retraction of the tonofilaments … while most of desmosomes remain intact” (step 3); and “collapse of the cytoskeleton and tearing off desmosomes from the cell membrane” (step 4) (37). Another recent finding supporting the apoptolysis model, where p38 phosphorylation is presumed to have a central role (Figure 1), comes from the observation that hampered DSG trans-interaction does not result in keratinocyte dissociation when p38 MAPK signaling is inhibited (46). Very recently, in fact, it has been shown that blockage of p38 MAPK prevents PV-IgG-induced blistering (47) and PF-IgG-induced desmosomal changes (48) both in human skin.

**Figure 2:**
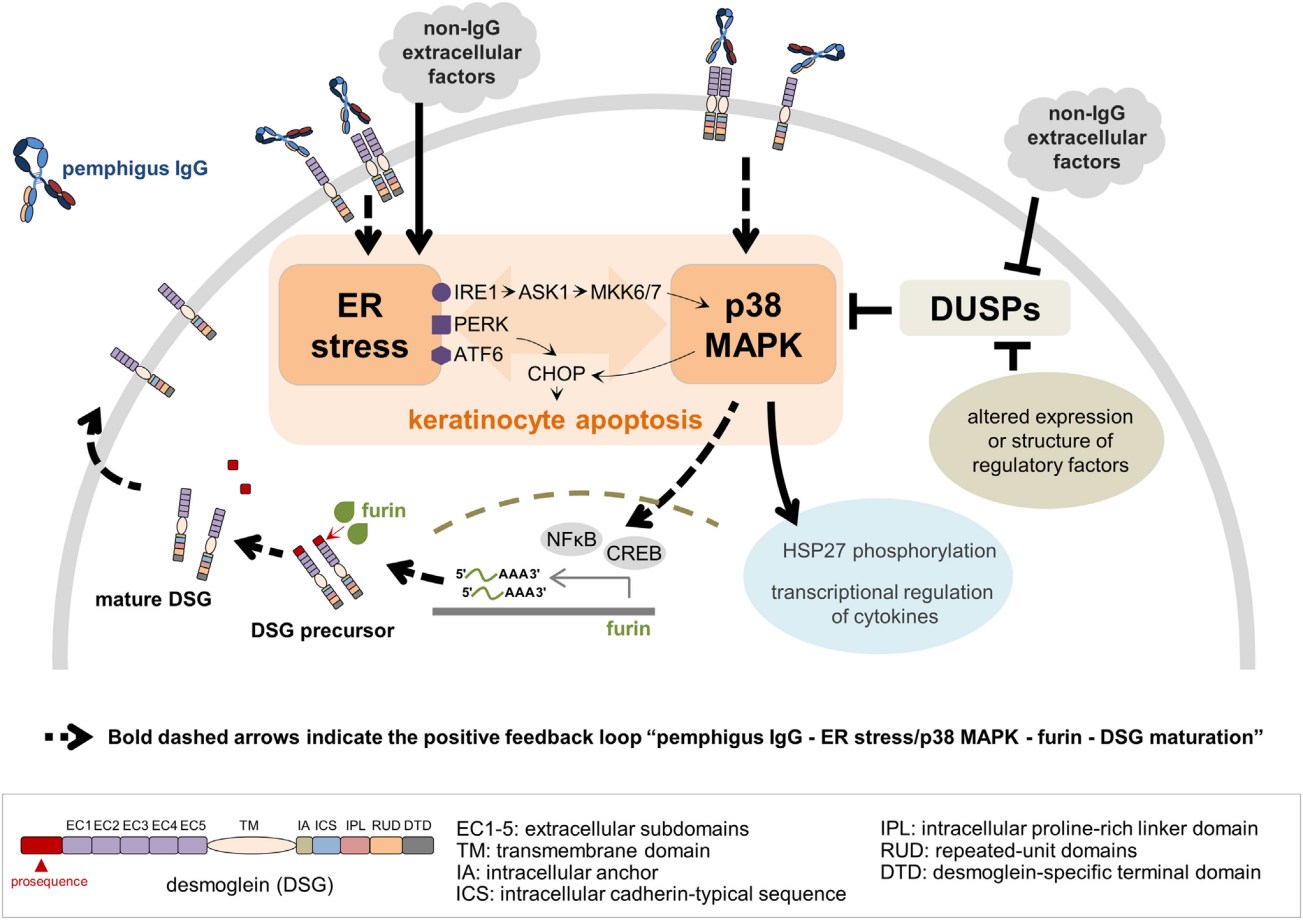
A model for a crosstalk between endoplasmic reticulum (ER) stress and p38 mitogen-activated protein kinase (p38 MAPK) pathway in the context of pemphigus. Both pemphigus IgG and non-IgG extracellular factors lead to ER stress resulting in C/EBP-homologous protein (CHOP) induction *via* protein kinase R-like ER kinase (PERK) and activating transcription factor 6 (ATF6). ER stress activates p38 MAPK through the inositol-requiring kinase 1 (IRE1)-apoptosis signal-regulating kinase 1 (ASK1)-MKK6/7 signaling pathway, and CHOP is activated by p38 MAPK. Pemphigus IgG binding preferentially to mature desmoglein (DSG) 1 and/or 3 activates the p38 MAPK pathway, which in turn induces ER stress. Dual-specificity phosphatases (DUSPs), negative regulators of p38 MAPK activation, can be targeted by either non-IgG extracellular factors or intracellular regulatory factors, such as RNAs and proteins, with altered expression or structure. ER stress and p38 MAPK play a critical role in keratinocyte apoptosis, heat shock protein 27 (HSP27) phosphorylation and transcriptional regulation of cytokines. In addition, activation of nuclear factor kappa-light-chain-enhancer of activated B cells (NFκB) and cAMP-responsive element binding protein (CREB) by p38 MAPK signaling pathway positively regulates *FURIN* transcription, which ultimately facilitates the DSG maturation process. As more mature DSG becomes available on the keratinocyte’s plasma membrane, the entire process restarts, characterizing the positive feedback loop.

More sophisticated studies have corroborated the simpler understanding that pemphigus autoAb induce loss of cell adhesion through steric hindrance. In another ultrastructural inspection of pemphigus skin and mucosa, two interesting findings have been reported for the first time in PF: the lack of desmosomes surrounding spontaneous blisters; and blistering in the below-granular layers when force was applied (38). According to the authors, these observations best fit the non-assembly depletion hypothesis, which may be considered a complementation of the steric hindrance hypothesis (25). It has also been verified that, among isolated monoclonal antibodies (mAb) of a PF patient, the single DSG1-specific pathogenic mAb recognizes exclusively the mature form of DSG1 lacking the N-terminal prosequence, while those non-pathogenic mAb were able to bind preferably the precursor form. Among all mAb, only the pathogenic mAb showed binding to the mature DSG1 region thought to be responsible for DSG trans-interaction (49). DSG1 maturation has been known to be regulated by furin, a proprotein convertase, *via* proteolytic cleavage of the prosequence (49, 50). Transcription of *FURIN* can be positively regulated by nuclear factor kappa-light-chain-enhancer of activated B cells (NFκB) (51) and cAMP-responsive element-binding protein (52), both of which are activated by p38 MAPK signaling pathways (51, 53). ER stress, in turn, has been very recently associated to PV’s pathogenesis (54). Therefore, considering that ER stress seems to be involved in pemphigus’ pathogenesis, while being known as an activator of the p38 MAPK pathway, which in turn has been reported to lead to ER stress, we focus on the potential connection between these pieces by suggesting that they may be linked as a positive feedback loop (Figure 2).

## p38 MAPK Signaling Pathway in Pemphigus

The importance of the p38 MAPK pathway involvement in pemphigus pathogenesis has been consistently reported throughout the literature (28, 29, 44, 46–48) and extensively reviewed elsewhere (55). The observations that DSG3 and p38 MAPK are in close proximity and that plakoglobin, p38 MAPK, and DSG3 can be co-immunoprecipitated have suggested the existence of a signaling complex formed by these molecules and its importance for the anchorage of the desmosomal plaque to the keratinocyte cytoskeleton (Figure 1) (56). Interestingly, phosphorylation of p38 MAPK induced by incubation of cultured keratinocytes with PV IgG can take place as early as 15 min, corroborating DSG3 and p38 MAPK association (32). However, this same study showed that, for the majority of patient-derived PV IgG, phosphorylated p38 MAPK did not reach its peaks until after 240 min from incubation. Such peaks were observed after an important reduction of p38 MAPK and increase of EGFRK and SRC phosphorylation at 60 min. Comparable findings had been reported previously (30). Meanwhile, it has been documented that p38 knockdown seems to prevent loss of desmosomal DSG3 and exogenous p38 activation appears to induce DSG3 internalization, both in PV IgG-treated keratinocyte cultures (57). Therefore, the late p38 phosphorylation peak has been interpreted as a consequence of internalized and processed DSG3, which in turn would not be primary for the loss of keratinocyte adhesion in PV, but actually an enhancer for blistering through DSG3 endocytosis.

Nonetheless, it is conceivable that such late peaks of p38 phosphorylation represent the activation of distinct pathways that converge to p38 MAPK *de novo* engagement, as in a positive feedback loop. In fact, negative feedback mechanisms insure that MAPKs are not uninterruptedly active. This task is undertaken by dual-specificity phosphatases (DUSPs), proteins with precise phosphorylating and dephosphorylating functions and with discrete cell-type distribution and subcellular localization (58). DUSP1, also known as MAP kinase phosphatase 1 (MKP1), is a well-known regulator of p38 MAPK activation, which may in turn induce a DUSP1-dependent negative feedback (59, 60). Besides the conceivable existence of a positive feedback loop downstream of a p38 phosphorylation and dephosphorylation cycle by DUSPs, these are themselves potentially associated with autoimmune diseases. DUSP1, for example, is underexpressed in psoriatic skin lesions in comparison to their normal-appearing counterparts (61), and this is believed to contribute to the inflammatory condition observed in the disease. Such an assumption derives from reports indicating reduced production of cytokines when inhibiting signaling through MAPKs. The PV-associated interleukin (IL)-8, for instance, seems to be downregulated with MKP1-dependent inhibition of p38 MAPK (62, 63). Finally, given the limited repertoire of known p38 dephosphorylators, regulatory RNAs that putatively target DUSPs could be comprehensively validated as such. Specifically, pemphigus-overexpressed microRNAs (miRNAs) putatively targeting DUSPs could be validated in light of their role in fine-tuning gene expression at the posttranscriptional level. This approach would be of special importance for validating the existence of a positive feedback loop, because of the expected upregulation of DUSP expression downstream of p38 MAPK activation and the potential existence of an abnormal miRNA profile in pemphigus keratinocytes. Again, such abnormal profile could include upregulated miRNAs that target DUSPs’ messenger RNA, therefore interfering with the negative feedback between newly synthesized DUSPs and p38 MAPK.

## The Emerging Role of ER Stress in Pemphigus

In contrast with the well-established role of p38 MAPK in pemphigus, the importance of ER stress in the disease has not yet undergone scrutiny. However, a link between ER stress and apoptosis has been demonstrated. During ER stress, proapoptotic transcription factor C/EBP-homologous protein (CHOP, or DNA damage-inducible transcript 3, DDIT3) is induced by PKR-like endoplasmic reticulum kinase (PERK) and activating transcription factor 6 (ATF6) on ER membranes. Inositol-requiring kinase 1 (IRE1α) on ER membranes is also activated by ER stress resulting in activation of p38 MAPK *via* apoptosis signal-regulating kinase 1 (ASK1) and MKK6/7. p38 MAPK in turn activates CHOP (Figure 2) [reviewed in Ref. (64)]. Engagement of p38 MAPK and JNK pathways were reported to take place initially after stimulation of Epstein–Barr virus-transformed B cells with anti-CD70—a treatment that leads to ER stress-mediated apoptosis of these cells—while inhibition of both pathways blocked upregulation of ER stress markers, such as CHOP (65). Also, p38 MAPK can function as an upstream inducer of ER stress (66, 67). This is indicative of the existence of an ER stress response *via* p38 MAPK and JNK pathways, suggesting that at least ER stress and p38 MAPK might be connected by a two-way route (Figure 2).

Some studies have investigated the involvement of ER stress in pemphigus pathogenesis. The ER stress pathway represented by the activation of PERK was shown to be engaged when exposing cultured keratinocytes to PV serum (68), which had been previously shown to upregulate PERK (69). Moreover, downregulation of PERK expression through small interfering RNA restricted the changes in keratinocyte cell cycle and viability typically observed after treatment of these cells with pemphigus serum (68). Interestingly, PERK phosphorylation can occur independently of PV IgG, i.e., when treating keratinocytes with total or Ig-depleted PV serum (68). However, by looking at the isolated effects of both anti-DSG1 and anti-DSG3 PV autoAb on ER stress induction, it was also reported that overexpression of CHOP may be anti-DSG1 dependent (54). These apparently conflicting results might be interpreted in light of the different intracellular signaling events triggered by the heterogeneous autoAb profiles of pemphigus patients (36). Moreover, in contrast with DSG3, it is unknown whether DSG1 is in association with p38 MAPK or not. Thus, the specific signaling complexes formed by both of these molecules could also explain such results.

Besides stimulating or being stimulated by the p38 MAPK pathway, ER stress could be contributing to the activation of HSP27 in pemphigus. Phosphorylation of this protein, known to occur in pemphigus and downstream of p38 MAPK and MAPK-activated protein kinase 2 (MK2, or MAPKAPK2) (70), has been reported to be induced by ER stress (71). Although environmental factors may play an important role in the pathogenesis and course of pemphigus, the contribution of viral infections to the disease remains unclear (72). Nonetheless, it has been reported that a hepatitis B virus envelope protein is capable of activating the p38 MAPK and NFĸB pathways in an ER stress-dependent manner (73), being therefore an example of how viral factors could directly contribute to a connected ER stress induction and p38 MAPK activation. Altogether, by adding ER stress to the understanding of pemphigus pathogenesis, an entirely new set of hypotheses and connections can be made in the context of the signaling pathways activated in the disease. Hence, we have put together a model in which ER stress has a potential central role in pemphigus (Figure 2). In summary, we suggest that ER stress may be triggered more directly by non-IgG factors, secondarily by anti-DSG1 autoAb—given the anti-DSG1-dependent induction of ER stress found by Mihailidou and collaborators (54)–or indirectly by pemphigus IgG *via* p38 phosphorylation. Once ER stress has been triggered, it can act as positive regulator of p38 phosphorylation. In addition, we suggest that pemphigus patients may produce factors that also, directly or indirectly, downregulate DUSP levels and act in conjunction with ER stress, for example, to allow for a secondary, but strong p38 MAPK engagement.

## Concluding Remarks

The existence of consistent data favoring either one of the molecular mechanisms explaining the loss of cell adhesion in pemphigus is consistent with its intricate pathogenesis. Indeed, histopathology may develop as a consequence of anti-DSG antibodies, both, impairing DSG trans-interaction through steric hindrance and subsequently transducing intracellular signals leading to keratinocyte apoptosis. However, non-IgG factors may also contribute to histopathology by inducing additional pathways, including ER stress, which may in turn activate the p38 MAPK signaling pathway of great importance in pemphigus. By connecting both, the ER stress and the p38 MAPK pathway, we put in perspective a potential positive feedback loop between these events in which, in an IgG-dependent manner, p38 MAPK activation leads to ER stress, which in turn stimulates p38 phosphorylation. We also suggest that, independently of autoAbs, i.e., also through factors such as viral particles, cytokines, metabolites, and/or regulatory RNAs and proteins, ER stress would primarily induce p38 MAPK activation, which would then prompt the positive feedback loop through the same intracellular signaling cascades. Finally, this is suggestive of a central role for ER stress in pemphigus pathogenesis and, by bringing this to light, we hope to inspire researchers in the field to deepen the understanding of this life-threatening disease.

## Author Contributions

All authors contributed substantially to the conception of the work. GC and JP drafted the work. RL and MP-E revised the work critically for intellectual content. All authors approved the final version of the work. All authors agreed to be accountable for all aspects of the work.

## Conflict of Interest Statement

The authors declare that the research was conducted in the absence of any commercial or financial relationships that could be construed as a potential conflict of interest.
